# A Comprehensive Analysis of Alterations in DNA Damage Repair Pathways Reveals a Potential Way to Enhance the Radio-Sensitivity of Esophageal Squamous Cell Cancer

**DOI:** 10.3389/fonc.2020.575711

**Published:** 2020-10-16

**Authors:** Guangchao Wang, Shichao Guo, Weimin Zhang, Zhangfu Li, Jiancheng Xu, Dan Li, Yan Wang, Qimin Zhan

**Affiliations:** ^1^State Key Laboratory of Molecular Oncology, National Cancer Center/National Clinical Research Center for Cancer/Cancer Hospital, Chinese Academy of Medical Sciences and Peking Union Medical College, Beijing, China; ^2^Key Laboratory of Carcinogenesis and Translational Research (Ministry of Education/Beijing), Laboratory of Molecular Oncology, Peking University Cancer Hospital & Institute, Beijing, China

**Keywords:** DNA damage repair pathways, esophageal squamous cell cancer, homologous recombination, non-homologous end joining, mirin, NU7441, radio-sensitivity

## Abstract

Esophageal squamous cell cancer (ESCC) is a common malignancy with a poor 5-year overall survival in China. Altered DNA damage repair (DDR) pathways are associated with a predisposition to cancer and contribute to therapeutic response and resistance in cancers. However, alterations of DDR pathway genes in ESCC are still largely unknown. In this study, we employed genome sequencing data of 192 samples, comparative genomic hybridization data of 123 cases, and gene expression microarray data of 119 patients to firstly perform a comprehensive analysis of the gene alterations of 7 DDR pathways in ESCC. Gene mutations and copy number variations (CNVs) were observed in all 7 DDR pathways, and especially, CNVs were the dominant alteration types. Compared with other pathways, two DNA double-strand break (DSB) repair pathways homologous recombination (HR) and non-homologous end joining (NHEJ), carried significant gene mutations and CNVs especially gene amplifications. Most genes including *RAD54B*, *NBS1*, *RAD51B*, and *PRKDC* were significantly amplified and over-expressed in ESCC. Amplification and high expression of DSB repair pathway genes were associated with poorer overall survival. Gene set variation analysis further showed that DSB repair pathways were up-regulated in ESCC. Besides, we firstly demonstrated that combination of mirin and NU7441, two inhibitors for HR and NHEJ respectively, with ionizing radiation treatment significantly enhanced DSBs, reduced clonogenic cell survival, inhibited cell proliferation, and promoted cell apoptosis in ESCC cells with DSB pathway gene amplification. These findings suggest that DSB repair pathways were significantly altered in ESCC and inhibiting DSB repair pathways might enhance the radio-sensitivity of ESCC with DSB repair up-regulation.

## Introduction

Esophageal cancer, principally comprising of two pathological types: esophageal squamous cell cancer (ESCC) and esophageal adenocarcinoma, is a global problem and the sixth leading cause of cancer mortality annually worldwide. The overall 5-year survival of patients with esophageal cancer ranges from 15 to 25%. ESCC accounts for 70% of cases of esophageal cancer globally and is the dominant type of esophageal cancer in China (1, 2). Recently, the diagnosis and treatment of ESCC have been improved, but the prognosis is still poor (1). The underlying mechanisms involved in tumorigenesis and progression of ESCC remain much less explored.

DNA damage repair (DDR) genes have crucial roles in maintaining genomic stability of human cells. According to biochemical and mechanistic criteria, DDR genes can be grouped into seven main functional pathways. Base excision repair (BER) and nucleotide excision repair (NER) are involved in DNA base damage repair, while mismatch repair (MMR) mainly corrects base mis-pairs. Homologous recombination (HR) and non-homologous end joining (NHEJ) are two pathways which contribute to DNA double-strand break (DSB) repair. In addition, the Fanconi anemia (FA) pathway is associated with the repair of DNA inter-strand crosslinks in the genome, and specialized DNA polymerases in trans-lesion synthesis (TLS) pathway synthesize DNA to bypass unrepaired DNA lesions (3, 4). Dysregulation of DDR pathways is an important determinant of cancer risk, progression, and therapeutic response (4). Up-regulation of DDR pathways are linked to cause resistance to DNA-damaging radiotherapy and chemotherapy. Especially, activation of DSB repair genes is one of the reasons for cancer radio-and chemo-resistance (4–11).

In ESCC, polymorphisms of BER genes were reported to be probably associated with the susceptibility to ESCC (12, 13). Genetic variants in NER genes were linked to exert an impact on survival outcomes of Chinese ESCC patients (14, 15). Moreover, genetic polymorphisms of *XRCC6* and *XRCC5*, two genes in NHEJ pathway, were related to higher risk of ESCC (16). In addition, promoter hypermethylation of the MMR gene *MLH1*, which is important for maintenance of genomic stability, may be a predictor of prognosis for male ESCC patients (17). However, the genetic alterations of DDR pathway genes in ESCC remain to be further investigated.

In the present study, we employed data from previously published studies to perform a comprehensive analysis of genetic alterations of DDR pathway genes in ESCC. Two DSB repair pathways, HR and NHEJ, showed significant gene mutations and amplifications. We investigated the gene expression profile of HR and NHEJ pathways with GSE53624 dataset, and found that most of genes were over-expressed in ESCC. Then, gene set variation analysis (GSVA) was conducted to analyze the pathway activity changes of HR and NHEJ, and DSB repair pathways were observed to be up-regulated in ESCC. We finally investigated the effect of combination of mirin and NU7441 with ionizing radiation (IR) treatment on ESCC cell phenotypes, and found that mirin and NU7441 could enhance the radio-sensitivity of ESCC cells with DSB pathway gene amplification. These findings suggest that alterations of DSB repair pathways might be involved in ESCC radio-resistance, and mirin and NU7441 might have potential application in ESCC treatment.

## Materials and Methods

### Data Collection and Processing

**Figure 1:**
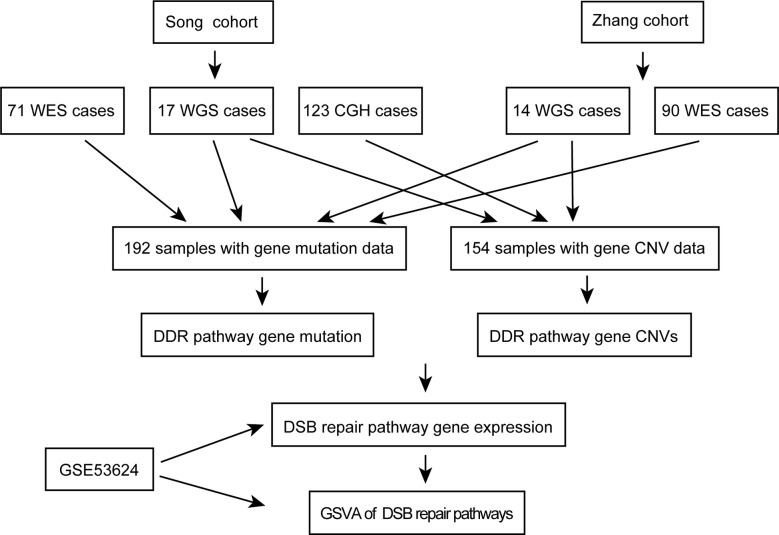
The workflow of data analysis in this study.

A workflow was designed to identify the gene alterations in DDR pathways (**Figure 1**). Our group previously published two studies to identify genomic alterations including gene mutations and copy number variations (CNVs) in ESCC (18, 19), and these two datasets were used to identify genomic alterations of DDR pathway genes. Two ESCC cohorts consist of a total of 262 cases, including 161 from whole-exome sequencing (WES), 31 from whole-genome sequencing (WGS), and 123 from comparative genomic hybridization (CGH) analysis. The data processing has been described in previous studies (18, 19). As the sequencing of both Song and Zhang cohorts was conducted in BGI, and the data of two cohorts has similar sequencing depth and coverage, we integrated the gene mutation data of two cohorts. Besides, we combined the gene CNV data generated by WGS and CGH. Consequently, we obtained 192 ESCC samples with gene mutation data, and 154 ESCC cases with CNV data. The clinical characteristics of two groups of ESCC patients were summarized in **Table 1**.

**Table 1:** The clinical characteristics of esophageal squamous cell cancer (ESCC) patients in this study.

Clinical characteristics	Total cases (Mutation cohort)	Total cases (CNV cohort)	Total cases (GSE53624)
Gender			
Male	170	122	98
Female	22	32	21
Age	^a^		
	104 (>=59)	84 (>=58)	66 (>=59)
	87 (<59)	70 (<58)	53 (<59)
TNM stage			
I+II	106	86	53
III	86	68	66
N stage			
N = 0	102	79	54
N > 0	90	75	65
T stage			
T1+T2	48	31	28
T3+T4	144	123	91
Drinking status			
Drinker	17	33	74
Non-drinker	175	121	45
Smoking status			
Smoker	153	104	80
Non-smoker	39	50	39
Survival	^b^	^c^	
Status			
Death	84	73	73
Survival	84	78	46
Median survival time (days)	902	751	32.2 (months)

^a^Age information of one ESCC patient is missing.

^b^Survival information of 24 ESCC patients is missing.

^c^Survival information of three ESCC patients is missing.

Additionally, in order to analyze the mRNA expression of DSB repair pathway genes in ESCC, we downloaded GSE53624 dataset, the mRNA expression profile of paired cancer, and adjacent normal tissues from 119 ESCC patients (20), from GEO (Gene Expression Omnibus, https://www.ncbi.nlm.nih.gov/geo/) database. To extract gene expression information of DDR genes in GSE53624, we re-annotated probes from Agilent-038314 CBC Homo sapiens lncRNA + mRNA microarray V2.0 platform (https://www.ncbi.nlm.nih.gov/geo/query/acc.cgi?acc=GPL18109). Human protein-coding transcript sequences (release 29) were downloaded from GENCODE (https://www.gencodegenes.org/) database. All probes in Agilent-038314 platform were then re-annotated as follow: 1, All probes sequences were aligned to human protein-coding transcript sequences with BLASTN. 2, The probes that were matched to one transcript or multiple transcripts from same genes were reserved. 3, The max expression value of multiple probes that were mapped to the same gene was calculated to represent the expression level of the gene. The clinical characteristics of 119 ESCC patients in GSE53624 were summarized in **Table 1**.

### Cell Lines

The human ESCC cell lines YES2 and KYSE30 were obtained from Y. Shimada’s lab in Kyoto University. YES2 and KYSE30 cells were cultured in Roswell Park Memorial Institute (RPMI) 1640 medium (Gibco, Thermo Fisher Scientific, USA) supplemented with 10% fetal bovine serum (FBS), and were grown at 37°C in humidified air with 5% CO_2_. The source of YES2 and KYSE30 cell lines have been recently authenticated and tested for mycoplasma contamination, and no contamination was found.

### Gene Set Variation Analysis

GSVA, using a nonparametric approach to transform a gene-by-sample matrix into a gene set-by-sample matrix, facilitates to determine the variation of pre-defined gene set activities over the samples based on gene expression data (21). Expression values of DSB repair pathway genes were used to perform GSVA *via* R “GSVA” package with the following parameters: method = “gsva,” mx.diff = “TRUE,” and kcdf = “Gaussian.”

### Immunofluorescence Analysis of γ-H2AX Expression

A total of 1×10^4^ YES2 and KYSE30 cells were seeded into confocal dishes for 24 h prior to treatment with mirin (Selleckchem, Houston, TX, USA) (50 µM) and NU7441 (Selleckchem, Houston, TX, USA) (5 µM). After treatment with inhibitors for 1 h, cells were exposed to 6 Gy of IR. Then, cells were cultured with inhibitors for 24 h. Subsequently, cells were fixed with 4% paraformaldehyde in phosphate-buffered saline (PBS) for 10 min at room temperature, then were permeabilized with PBST (0.5% Triton X-100 in PBS) for 10 min on ice. Nonspecific binding was blocked with 1% bovine serum albumin (BSA) in PBST for 30 min. Then, the cells were incubated in the diluted antibody against γ-H2AX (ab26350, Abcam) in 1% BSA (1:200) in a humidified chamber for overnight at 4°C and followed by incubation with Alexa Fluor 488-conjugated secondary antibody (ZSGB-BIO, Beijing, China) in PBS for 1 h at room temperature in the dark. Immunofluorescence images were taken by using laser-scanning confocal microscope (Leica Microsystems Heidelberg GmbH, Am Friedensplatz 3, Germany).

### Clonogenic Assay

To assess how combination of inhibitors with IR treatment affects clonogenic cell survival, YES2, and KYSE30 cells were seeded into six-well plates at a density of 1,500 and 1,000 cells per well, respectively. The cells were incubated for 10 days. Then, cells were treated with mirin (50 µM) and NU7441 (5 µM) for 1 h and irradiated afterward once with 6 Gy. After incubation with inhibitors for an additional 3 days, the cells were fixed with methanol for 5 min, and stained with 0.05% crystal violet (Sigma Chemical Company, St. Louis, MO, USA) for 5 min. Colonies were counted by using ImageJ 1.52V software.

### Cell Proliferation Assay

To examine the effect of combination of inhibitors with IR treatment on cell proliferation, YES2 and KYSE30 cells were seeded into 96-well plates at a density of 5,000 cells per well for 24 h. Then, cells were incubated with mirin (50 µM) and NU7441 (5 µM) for 1 h, followed by being exposed to 6 Gy of IR. Subsequently, the optical density (OD) value at 490 nm was detected after 0, 24, 48, 72, and 96 h with a microplate reader (iMark™, BIO-RAD) after treatment with [3-(4,5-dimethylthiazol-2-yl)-5-(3-carboxymethoxyphenyl)-2-(4-sulfophenyl)-2H-tetrazolium, inner salt] (MTS) (Promega) solution (10% MTS in RPMI 1640 medium) for 1 h. The experiment was repeated three times, and the ratio of OD value (hours 24–96) to the average value of 0 h was calculated and plotted as MTS curves.

### Cell Apoptosis Assay

A total of 2×10^5^ YES2 and KYSE30 cells were seeded into 6 cm dishes and cultured for 24 h. Subsequently, cells were treated with mirin (50 µM) and NU7441 (5 µM) for 1 h, and were then exposed to 6 Gy of IR. After being cultured with inhibitors for 24 h, cells were collected and stained with annexin V and propidium iodide (PI) according to the manufacturer’s instruction provided in Annexin V-FITC/PI apoptosis assay kit (NEOBIOSCIENCE, Shenzhen, China). Flow cytometry (BD LSR) was used to determine the percentage of apoptotic cells.

### Statistical Analysis

All statistical tests and graphing were performed by R 3.6.0 and GraphPad Prism 7.0. All of the experiments in this study were independently performed in triplicate, and the data was presented as mean ± standard deviation (S.D.). Fisher’s exact test was applied to gene mutation enrichment analysis. Survival curves were performed by Kaplan-Meier method, and the differences between the curves were estimated by log-rank test. Welch’s unequal variances *t*-test was used to compare the GSVA scores in ESCC and normal samples, and to analyze the correlations between the GSVA scores and clinical characteristics of ESCC patients. ESCC patients were divided into two groups (high and low groups) according to the median value of gene expression or GSVA scores, and survival analysis was conducted by Kaplan-Meier method. Besides, the correlations between gene expression and clinical characteristics of ESCC patients were analyzed with Fisher’s exact test. The other statistical analyses were performed with Student’s *t*-test. Each *P* was two-sided, and *P* < 0.05 was considered statistically significant.

## Results

In order to investigate the landscape of genetic alterations in DDR pathways, we defined a “core DDR” gene set of 79 DNA repair pathway-specific genes (genes annotated to more than one specific DDR pathway were not included), encompassing 7 major DDR pathways: BER, NER, MMR, HR, NHEJ, FA, and TLS (**Table 2**) (3, 22).

**Table 2:** Gene lists of seven DNA damage repair (DDR) pathways.

BER	NER	MMR	HR	NHEJ	FA	TLS
*UNG*	*XPC*	*PMS2*	*XRCC3*	*XRCC6*	*UBE2T*	*SHPRH*
*TDP1*	*XPA*	*PMS1*	*XRCC2*	*XRCC5*	*FANCM*	*REV3L*
*TDG*	*POLE3*	*MSH6*	*TOP3A*	*XRCC4*	*FANCL*	*REV1*
*POLB*	*POLE*	*MSH3*	*SLX1A*	*PRKDC*	*FANCI*	*POLQ*
*PARP1*	*ERCC6*	*MSH2*	*SHFM1*	*POLM*	*FANCD2*	*POLN*
*FEN1*	*ERCC5*	*MLH3*	*RBBP8*	*POLL*	*FANCC*	*POLK*
*APEX2*	*ERCC4*	*MLH1*	*RAD52*	*NHEJ1*	*FANCB*	*POLH*
*APEX1*	*ERCC2*	*EXO1*	*RAD51*	*LIG4*	*FANCA*	*POLI*
*LIG1*	*ERCC1*		*RAD50*	*TP53BP1*	*FANCE*	
*LIG3*	*CUL5*		*PALB2*		*FANCF*	
			*NBS1*		*FANCG*	
			*MUS81*			
			*MRE11*			
			*GEN1*			
			*EME1*			
			*BRIP1*			
			*BRCA2*			
			*BRCA1*			
			*BLM*			
			*BARD1*			
			*RAD51B*			
			*RAD54B*			
			*WRN*			

### DNA Damage Repair Pathway Genes Were Mutated in Esophageal Squamous Cell Cancer

**Figure 2:**
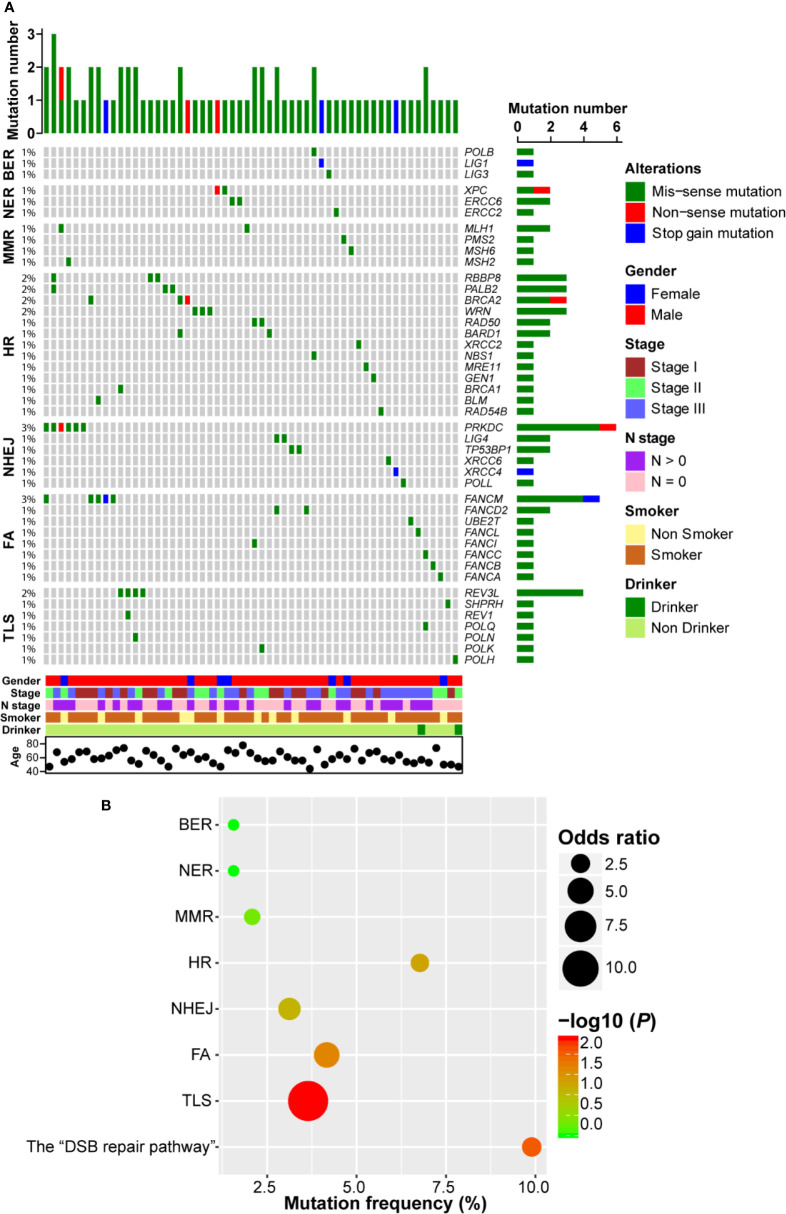
DNA damage repair (DDR) pathway genes were mutated in esophageal squamous cell cancer (ESCC). **(A)** A complex heatmap that shows the non-silent gene mutation profile in DDR pathways (genes and samples with no gene mutations are removed). The top panel presents the number of gene mutations in each of ESCC samples, and the right panel shows the number of gene mutations in each gene. **(B)** A bubble plot that depicts the gene mutation enrichment analysis result of DDR pathways.

Firstly, we investigated the non-silent somatic mutation profile of DDR pathway genes in ESCC. As shown in **Figure 2A**, we observed that gene mutation occurred in all 7 DDR pathways and 44 genes were mutated. There were three genes (*POLB*, *LIG1*, and *LIG3*) mutated in BER pathway, each of which only carried one mutation. In NER pathway, both *XPC* and *ERCC6* had two mutations and *ERCC2* had one mutation. Similarly, three genes (*PMS2*, *MSH2*, and *MSH6*) in MMR pathway each had one mutation event and *MLH1* was mutated in two samples. In HR pathway, *BRCA1/2* showed four mutation events (2.1%, 4/192). We also observed that MRE11-RAD50-NBS1 (MRN) complex genes, which play important roles in the sensing, processing and repair of DSBs (23), were mutated in four ESCC patients. Besides, *RBBP8*, *PALB2*, *WRN*, and *BARD1* were mutated in more than one ESCC case. In NHEJ pathway, another pathway involved in repairing DSBs, *PRKDC*, which encodes the catalytic subunit of the DNA-dependent protein kinase (DNA-PK), carried the most frequent mutations (3.1%, 6/192). Furthermore, both *TP53BP1* and *LIG4* had two mutations. *FANCM* was the highest frequently mutated gene of FA pathway (2.6%, 5/192), and *REV3L* was the most frequently (2.1%, 4/192) mutated gene in TLS pathway. Interestingly, we observed that most ESCC samples only had one DDR gene mutation, indicating a mutually exclusive tendency.

In order to further evaluate the significantly mutated DDR pathways, we performed pathway enrichment analysis. As shown in **Figure 2B**, BER, NER, and MMR pathway mutations were not enriched. Gene mutations in FA and TLS pathways were significantly enriched (*P* = 0.022 and *P* = 0.004, respectively). Similarly, HR and NHEJ pathways tended to be enriched in ESCC (*P* = 0.05 and *P* = 0.08, respectively). We combined HR and NHEJ as the “DSB repair pathway” and conducted enrichment analysis. The result showed that the “DSB repair pathway” mutation was significantly enriched in ESCC (*P* = 0.009; **Figure 2B**).

### DNA Damage Repair Pathway Genes Had Notable Copy Number Variations in Esophageal Squamous Cell Cancer

**Figure 3:**
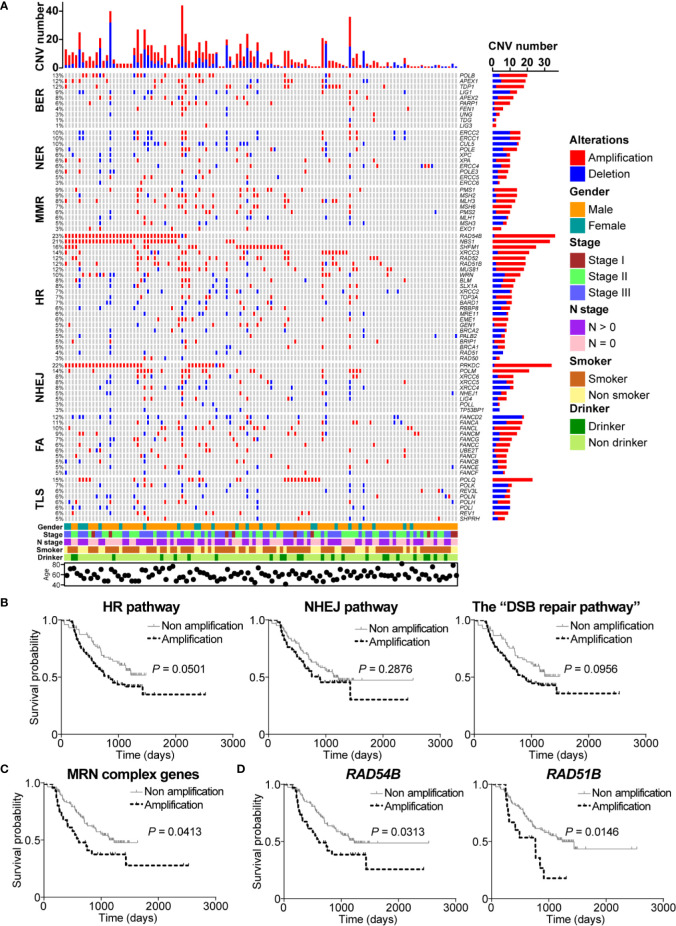
DNA damage repair (DDR) pathway genes had significant copy number variations (CNVs) in esophageal squamous cell cancer (ESCC). **(A)** A complex heatmap that shows the gene CNV profile in DDR pathways (samples with no CNVs are removed). The top panel presents the number of gene CNVs in each of ESCC samples, and the right panel shows the number of gene CNVs in each gene. **(B)** Amplification of homologous recombination (HR), non-homologous end joining (NHEJ), and the “DSB repair pathway” was associated with poorer overall survival. The amplification of MRE11-RAD50-NBS1 (MRN) complex genes **(C)**, *RAD54B*, and *RAD51B* **(D)** was related to shorter overall survival.

Next, we identified the CNV profile of DDR pathway genes in ESCC. As the complex heatmap in **Figure 3A** shown, CNVs occurred in all of DDR pathway genes, and 74.7% (115/154) of ESCC samples possessed at least one gene CNV in DDR pathways. In BER pathway, genes were mainly amplified rather than deleted. Both *POLB* and *TDP1* were amplified in 10.4% (16/154) of cases and the incidence of amplification of *APEX1/2* was 14.3% (22/154). Besides, *PARP1* revealed 5.8% (9/154) of amplification frequency and 0.6% (1/154) of deletion frequency. On the contrary, some genes in NER pathway had more deletions instead of amplifications. *CUL5* had the highest deletion frequency (9.1%, 14/154), and *ERCC1* and *ERCC2* had the same CNV profile (3.9% of amplification and 6.5% of deletion) due to proximal genomic location. Gene polymorphisms of *XPA* and *XPC* were reported to be associated with increasing risk of ESCC (15). We observed that *XPA* was amplified in 8 ESCC patients (5.2%) and deleted in 2 ESCC cases (1.3%), whereas the incidences of amplification and deletion of *XPC* were 1.3% (2/154) and 5.2% (8/154), respectively. *MLH1*, one of the important genes in MMR process, was observed to be deleted in 5.2% (8/154) of ESCC cases. Other genes in MMR pathway mainly had more amplifications than deletions.

Interestingly, we found that most of HR pathway genes were mainly amplified. The most amplified gene was *RAD54B* (22.7%, 35/154) which is related to multiple cancers (24–26). However, how *RAD54B* links to ESCC is unclear. *NBS1*, which encodes one of important proteins in MRN complex, was amplified in 21.4% (33/154) of ESCC patients. Cheng and colleagues reported that *XRCC3* was up-regulated in ESCC and was a potential target to improve the radiotherapy effect of ESCC (8). We observed that *XRCC3* was amplified in 11.7% (18/154) of ESCC cases, indicating that up-regulation of *XRCC3* might be due to gene amplification. Other genes such as *SHFM1*, *RAD52*, *RAD51B*, and *MUS81* were also remarkably amplified in ESCC with the frequency 16.2% (25/154), 12.3% (19/154), 11% (17/154), and 10.4% (16/154), respectively. In NHEJ pathway, *PRKDC* was one of significantly amplified DDR pathway genes with the amplification frequency 21.4% (33/154). Another notably amplified gene was *POLM* (13.6%, 21/154), which has not been studied in cancers yet. Besides, *XRCC6* had more amplification events (5.8%, 9/154) than deletions (1.9%, 3/154) (**Figure 3A**).

Additionally, *FANCD2*, carrying the most CNVs in FA pathway, was mainly deleted in 11% (17/154) of ESCC cases. Conversely, *FANCL*, *FANCM*, *FACC*, *UBE2T*, and *FANCI* obviously had more amplification events. In TLS pathway, although *POLQ* did not have significant mutation events, this gene was obviously amplified in ESCC (14.9%, 23/154). Similarly, the main CNV type of *POLH* and *REV1* was deletion. *REV3L*, the most mutated gene in TLS pathway, carried 8 (5.2%) amplification events (**Figure 3A**).

Given the fact that HR and NHEJ, the DSB repair pathways, had significant gene amplifications, we were interested to investigate the correlation between amplification of DSB repair pathways and clinical characteristics of ESCC patients. Survival analysis showed that ESCC samples with amplification of HR or NHEJ pathway had shorter overall survival (*P* = 0.0501 and *P* = 0.2876, respectively; **Figure 3B**). Similarly, we found that ESCC patients with the “DSB repair pathway” amplification had poorer overall survival (*P* = 0.0956; **Figure 3B**). Besides, MRN complex gene amplification was associated with shorter overall survival (*P* = 0.0413; **Figure 3C**). In addition, we performed survival analysis of DSB repair pathway genes with amplification frequency more than 10%. As presented in **Figure 3D**, amplification of *RAD54B* or *RAD51B* was related to poorer overall survival (*P* = 0.0313 and *P* = 0.0146, respectively).

### Double-Strand Break Repair Pathway Genes Were Up-Regulated in Esophageal Squamous Cell Cancer

**Figure 4:**
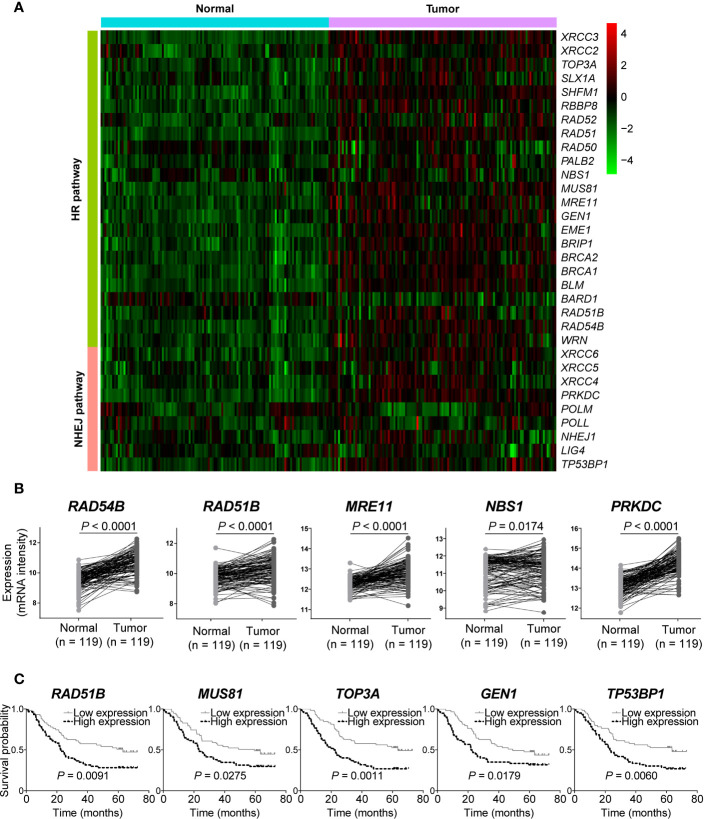
The messenger RNA (mRNA) expression level of double-strand break (DSB) repair pathway genes was up-regulated in esophageal squamous cell cancer (ESCC). **(A)** A heatmap that depicts the mRNA expression profile of DSB repair pathway genes in ESCC and normal tissues from GSE53624 dataset. **(B)** Student’s *t*-test analysis showed that *RAD54B*, *RAD51B*, *MRE11*, *NBS1*, and *PRKDC* were up-regulated in ESCC with statistically significant *P* values. ESCC patients were divided into two groups based on the median expression values of DSB repair pathway genes, and survival analysis was then performed. High expression of *RAD51B*, *MUS81*, *TOP3A*, *GEN1*, and *TP53BP1* was associated with poorer overall survival of ESCC patients **(C)**.

We next analyzed gene expression of DSB repair pathways in GSE53624 dataset. Interestingly, we observed that most of genes were up-regulated in ESCC samples compared with normal tissues (**Figure 4A**). Both *RAD54B* and *RAD51B* were significantly over-expressed in ESCC (*P* < 0.0001 and *P* < 0.0001, respectively; **Figure 4B**). Similarly, *MRE11* and *NBS1*, two MRN complex genes, and the most amplified NHEJ pathway gene *PRKDC* were also markedly up-regulated in ESCC (*P* < 0.0001, *P* = 0.0174, and *P* < 0.0001, respectively; **Figure 4B**).

We also investigated the correlations between expression of DSB repair pathway genes and clinical traits of ESCC patients. Survival analysis showed that high expression of five genes including *RAD51B*, *MUS81*, *TOP3A*, *GEN1*, and *TP53BP1* were related to shorter overall survival (*P* = 0.0091, *P* = 0.0275, *P* = 0.0011, *P* = 0.0179, and *P* = 0.0060, respectively; **Figure 4C**). **Table 3** summarized the correlation between gene expression and clinical characteristics. We found that gene expression of *RAD51B* and *TOP3A* was associated with TNM stage (*P* = 0.043 and *P* = 0.043, respectively). Besides, *XRCC5* expression was related to lymph node metastasis (*P* = 0.027).

**Table 3:** The correlations between gene expression of homologous recombination (HR) and non-homologous end joining (NHEJ) pathways and clinical characteristics of esophageal squamous cell cancer (ESCC) patients in GSE53624 dataset.

	Gender	Age	TNM stage	N stage	T stage	Drinking status	Smoking status
*XRCC3*	0.814	0.582	0.582	0.582	0.518	0.706	0.697
*XRCC2*	0.480	0.713	0.855	0.359	0.670	0.451	0.697
*TOP3A*	0.814	0.098	**0.043**	0.142	0.829	0.348	0.175
*SLX1A*	0.632	1.000	0.855	1.000	0.829	0.059	0.697
*SHFM1*	0.097	0.141	0.713	1.000	0.829	0.188	0.847
*RBBP8*	0.814	1.000	0.855	0.582	0.394	1.000	0.562
*RAD52*	1.000	0.855	0.359	0.464	1.000	0.706	0.697
*RAD51*	0.814	1.000	0.582	1.000	0.200	0.348	0.081
*RAD50*	0.632	0.462	0.855	0.272	0.829	0.573	0.847
*PALB2*	0.814	1.000	0.359	0.272	0.518	0.091	0.562
*NBS1*	0.632	0.141	0.462	0.855	1.000	0.573	0.333
*MUS81*	1.000	0.270	0.582	0.464	1.000	0.851	0.242
*MRE11*	0.632	0.713	0.855	0.714	0.394	0.451	0.697
*GEN1*	0.480	0.855	0.855	0.272	0.130	1.000	0.562
*EME1*	0.632	0.462	0.855	1.000	0.518	0.348	0.333
*BRIP1*	0.632	0.270	1.000	0.272	0.280	0.573	0.562
*BRCA2*	0.238	0.141	0.855	0.855	0.200	1.000	0.562
*BRCA1*	0.238	0.713	1.000	0.855	0.518	0.091	0.081
*BLM*	1.000	0.855	1.000	0.359	0.394	0.451	0.562
*BARD1*	0.814	0.855	0.199	0.066	1.000	1.000	0.847
*RAD51B*	1.000	0.359	**0.043**	0.142	1.000	0.348	0.847
*RAD54B*	0.480	1.000	0.270	0.855	0.518	0.573	**0.033**
*WRN*	0.814	0.713	0.855	0.272	0.518	0.091	**0.033**
*XRCC6*	0.337	0.359	0.582	0.714	1.000	1.000	1.000
*XRCC5*	0.632	1.000	0.582	**0.027**	0.280	0.573	0.175
*XRCC4*	1.000	0.582	0.582	0.464	1.000	0.851	0.847
*PRKDC*	0.814	1.000	0.855	0.142	0.280	0.573	0.081
*POLM*	0.480	0.270	0.359	0.714	1.000	0.851	1.000
*POLL*	1.000	0.855	0.359	0.272	0.829	0.573	0.847
*NHEJ1*	0.632	0.359	0.359	0.272	0.051	0.706	0.847
*LIG4*	0.337	0.141	0.582	1.000	0.200	1.000	0.242
*TP53BP1*	0.337	0.199	0.582	0.464	0.670	0.573	0.847

Statistically significant P values were in bold.

### Gene Set Variation Analysis Showed Up-Regulation of Double-Strand Break Repair Pathways in Esophageal Squamous Cell Cancer

**Figure 5:**
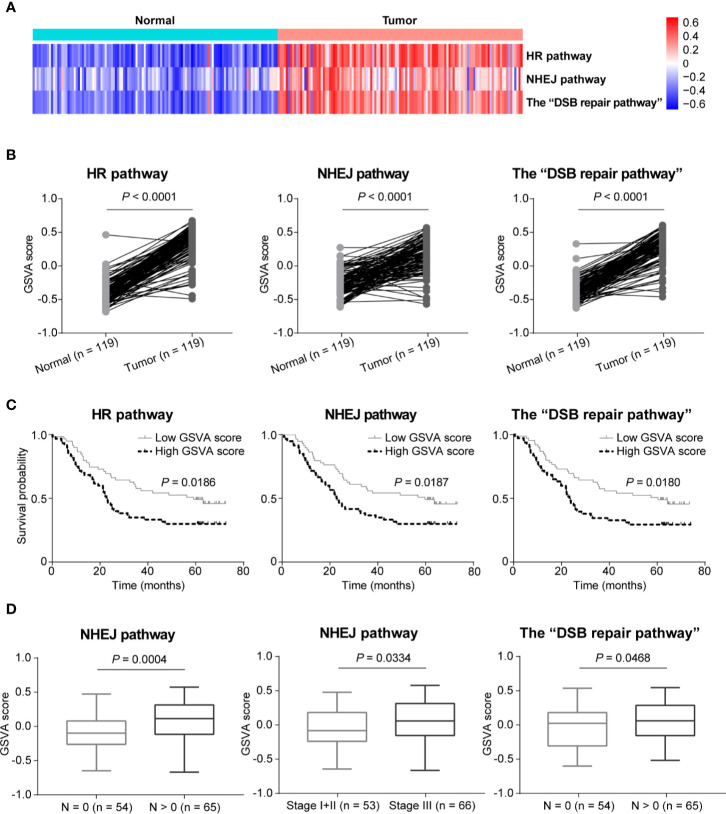
The activities of double-strand break (DSB) repair pathways were up-regulated in esophageal squamous cell cancer (ESCC) as determined by gene set variation analysis (GSVA). **(A)** A heatmap that shows the GSVA scores of homologous recombination (HR), non-homologous end joining (NHEJ), and the “DSB repair pathway” in each ESCC or normal sample. **(B)** Welch’s unequal variances *t*-test result showed that the activities of HR, NHEJ and the “DSB repair pathway” were significantly up-regulated in ESCC based on the GSVA scores. ESCC patients were divided into two groups based on the median GSVA scores of HR, NHEJ and the “DSB repair pathway” respectively, and survival analysis was then performed. High activities of HR, NHEJ, and the “DSB repair pathway” were associated with shorter overall survival **(C)**. **(D)** Compared to ESCC patients with N = 0, the GSVA scores of NHEJ and the “DSB repair pathway” were significantly higher in ESCC cases with N > 0. Besides, the GSVA scores of NHEJ in ESCC samples of stage III were significantly higher than ESCC patients of stage I and II.

In order to further compare the activities of DSB repair pathways between ESCC and normal tissues, we conducted GSVA based on expression of DSB repair pathway genes. We observed that pathway activities of HR, NHEJ and the combined “DSB repair pathway” were all significantly up-regulated in ESCC based on GSVA scores (*P* < 0.0001, *P* < 0.0001, and *P* < 0.0001, respectively; **Figures 5A, B**). Moreover, survival analysis showed that high pathway activities of HR, NHEJ, and the “DSB repair pathway” were associated with poorer overall survival (*P* = 0.0186, *P* = 0.0187, and *P* = 0.0180, respectively; **Figure 5C**). Besides, the GSVA scores of NHEJ pathway were higher in ESCC cases with lymph node metastasis (*P* = 0.0004; **Figure 5D**). Similarly, the GSVA scores of NHEJ pathway in ESCC stage III group were higher than stage I and II group (*P* = 0.0334; **Figure 5D**). The GSVA scores of the “DSB repair pathway” were obviously increased in ESCC cases with lymph node metastasis (*P* = 0.0468; **Figure 5D**).

### Combination of Mirin and NU7441 With Ionizing Radiation Treatment Significantly Enhanced DNA Double-Strand Breaks, Reduced Clonogenic Cell Survival, Inhibited Cell Proliferation, and Promoted Cell Apoptosis in Esophageal Squamous Cell Cancer Cells

Although radiotherapy is one of the effective treatments for ESCC, some ESCC patients often show no response or encounter adverse effects as a result of tumor radio-resistance (27, 28). As IR can induce a variety of DNA damages especially double-strand breaks, HR and NHEJ pathways play an important role in causing radio-resistance (5, 7, 29). In this study, we observed that HR and NHEJ pathway genes were significantly up-regulated in ESCC. Accordingly, we assumed that inhibition of HR and NHEJ pathways might enhance the radio-sensitivity of ESCC with DSB repair pathway up-regulation. In HR pathway, the MRN complex is essential for sensing and signaling from DNA double-strand breaks and promoting homology-dependent DNA repair (23). As mentioned above, two MRN complex genes *MRE11* and *NBS1* were up-regulated in ESCC. DNA-PK, a protein kinase complex composed of a Ku70/Ku80 heterodimer and a catalytic subunit encoded by *PRKDC*, plays a crucial role in facilitating NHEJ repair for DNA double-strand breaks and was identified as a potential anticancer target (30, 31). Similarly, over-expression of *PRKDC* was identified in ESCC. Therefore, mirin and NU7441, the highly potent and selective inhibitors for MRN complex and DNA-PK respectively (32–34), were utilized to assess whether inhibition of DSB repair pathways could improve the radio-sensitivity of ESCC cell lines with altered DSB repair pathways.

**Figure 6:**
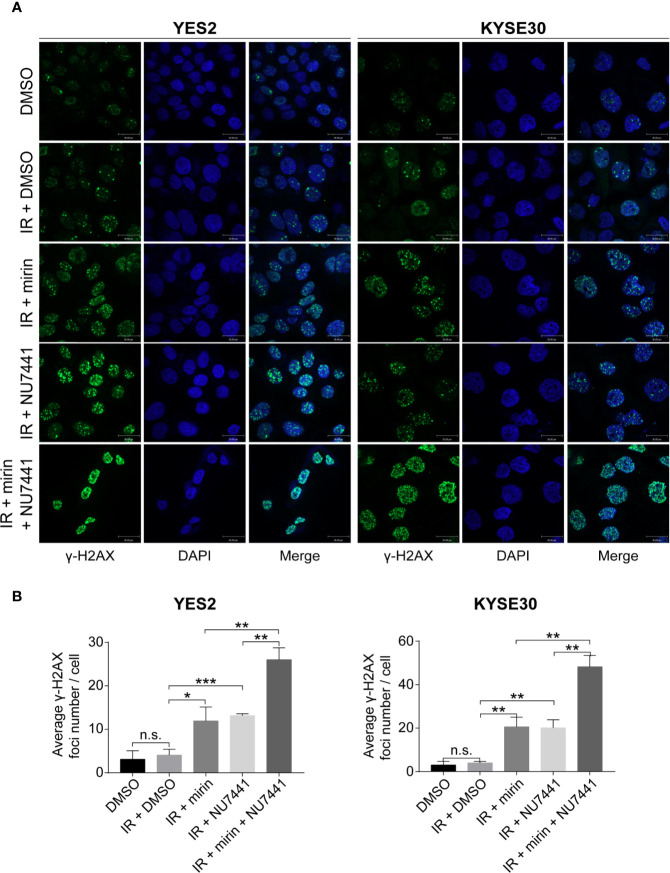
Combination of mirin and NU7441 with ionizing radiation (IR) treatment significantly enhanced double-strand breaks (DSBs) in esophageal squamous cell cancer (ESCC) cells. **(A, B)** DSBs were indicated by immunostaining with γ-H2AX. Combinations of mirin (50 µM) or/and NU7441 (5 µM) with IR (6 Gy) treatment significantly improved the number of γ-H2AX foci in both YES2 and KYSE30 cells. Scale bar = 30 µm. All the experiments were independently performed in triplicate. The error bars represent the standard deviation and *P* values were evaluated by Student’s *t*-test. *P ≤ 0.05, **P ≤ 0.01, ***P ≤ 0.001, n.s. *P* > 0.05.

We found that most DSB repair pathway genes were amplified in YES2 and KYSE30 cells according to the result of WGS on ESCC cell lines previously conducted in our laboratory (data not published). Therefore, we used YES2 and KYSE30 cells to investigate the effects of mirin and NU7441. We firstly tested if mirin and NU7441 could induce DSBs. As phosphorylation of H2AX (γ-H2AX) is a hall marker of DSBs (35), we conducted an immunofluorescence assay to determine the number of γ-H2AX foci after 24 h of treatment with IR and inhibitors in ESCC cells. The level of γ-H2AX had a little increasing following 6 Gy IR treatment alone, whereas both mirin and NU7441 enhanced γ-H2AX recruitment and combination of two inhibitors led to the higher level of γ-H2AX (**Figures 6A, B**), indicating that inhibiting DSB repair pathways could enhance IR-inducing DSBs in ESCC cells.

**Figure 7:**
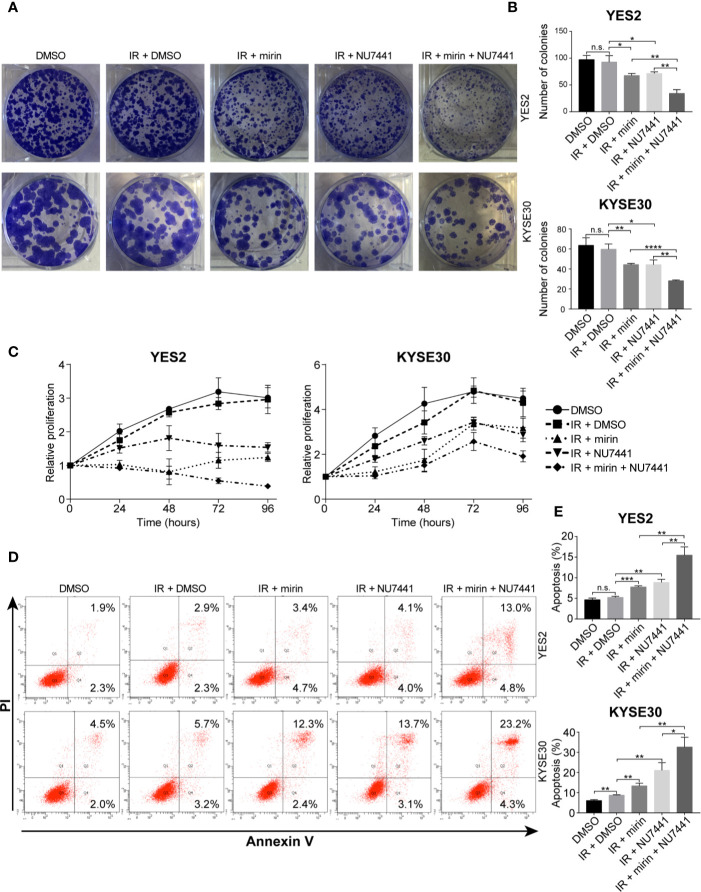
Combination of mirin and NU7441 with ionizing radiation (IR) treatment significantly reduced clonogenic cell survival, inhibited cell proliferation and promoted cell apoptosis in esophageal squamous cell cancer (ESCC) cells. **(A, B)** Clonogenic cell survival with combinations of inhibitors and IR (6 Gy) treatment was investigated by clonogenic assay. Combinations of mirin (50 µM) or/and NU7441 (5 µM) with IR treatment significantly reduced number of colonies of both YES2 and KYSE30 cells. **(C)** Cell proliferation was measured at 24, 48, 72, and 96 h after treatment with inhibitors and IR (6 Gy) by MTS assay. Combinations of mirin (50 µM) or/and NU7441 (5 µM) with IR treatment significantly inhibited proliferation of both YES2 and KYSE30 cells. **(D, E)** Flow cytometric analysis was applied to detect the effect of combinations of inhibitors and IR (6 Gy) treatment on cell apoptosis. Combinations of mirin (50 µM) or/and NU7441 (5 µM) with IR treatment significantly promoted cell apoptosis of both YES2 and KYSE30 cells. All the experiments were independently performed in triplicate. The error bars represent the standard deviation and *P* values were evaluated by Student’s *t*-test. *P ≤ 0.05, **P ≤ 0.01, ***P ≤ 0.001, ****P ≤ 0.0001, n.s. *P* > 0.05.

Next, to investigate the effect of mirin and NU7441 on clonogenic cell survival of ESCC cells, we conducted clonogenic assay. IR treatment alone did not have a significant influence on clonogenic survival of both YE2 and KYSE30 cells (**Figures 7A, B**). Combining IR with mirin or NU7441 treatment showed notable reduction in clonogenic survival of ESCC cells, and combination of mirin and NU7441 with IR treatment led to the lowest number of colonies in both cells (**Figures 7A, B**). We performed MTS assay in a 96 h interval to detect how mirin and NU7441 treatment affects cell proliferation. As shown in **Figure 7C**, compared with negative control, the proliferation of ESCC cells did not obviously decrease with IR treatment alone. Interestingly, we observed that combining IR with mirin or NU7441 showed significant inhibition of cell proliferation. Moreover, combination of two inhibitors presented the strongest inhibition ability (**Figure 7C**). Apoptosis is considered as one of the main forms of cell death induced by IR. We investigated the effect of IR, mirin and NU7441 on apoptosis in ESCC cells. Similarly, 6 Gy IR treatment alone had a little effect on promoting ESCC cell apoptosis (**Figures 7D, E**). The apoptosis rates were obviously increased in groups combining IR with mirin or NU7441 treatment (**Figures 7D, E**). Furthermore, the synergistic effect of mirin and NU7441 dramatically promoted cell apoptosis (**Figures 7D, E**).

## Discussion

Personalized care has become a key part of developing effective treatment guidelines for human cancer. One of the most important aspects of precision medicine in cancer is matching patients and treatments based on the genomic features of an individual and their tumor (36). As genomics-driven precision medicine extends beyond somatic mutations, comprehensive cancer sequencing to identify structural and copy number variations, as well as abnormal expression is becoming increasingly relevant to guide cancer therapy (37). Although diagnosis and treatment of ESCC have been improved, the prognosis is still poor. The development of ESCC is the result of a complex process with several steps implicated in multiple gene alterations (1, 2, 38). Thus, better patient stratification is needed to develop personalized treatment strategies for ESCC. Genomics-driven precision medicine may fulfill this urgent need.

One hallmark of cancer is genomic instability induced by various insults that lead to DNA damage (39). DDR plays a critical role on the protection of genomic stability to prevent from tumorigenesis. Alterations in DDR pathways play important roles in the development of cancers. In melanoma, gene up-regulation in DDR pathways is associated with tumor metastasis (40). DDR gene mutations were linked to immune-related gene expression in ovarian cancer and muscle invasive bladder cancer (41, 42). DDR was also reported to be involved in cancer metabolism. Activated DDR increases nucleotide synthesis and anabolic glucose metabolism, while reduces glutamine anaplerosis (43). Besides, up-regulated DDR pathways is one of important factors that trigger resistance to radiotherapy and chemotherapy (4–6). Therefore, identification of alterations in DDR pathways is helpful for better understanding the mechanisms of cancer progression. Moreover, targeting altered DDR pathways could be an effective way for cancer treatment (4, 5). Gene polymorphisms in BER, NER and NHEJ pathway genes have been reported to be related to higher risk of ESCC (12–16). However, the alterations in DDR pathways, including gene mutations, CNVs, and abnormal expression, are still largely unknown in ESCC, and how alterations in DDR pathways contribute to ESCC needs to be further explored.

In this study, we firstly performed a comprehensive analysis of genomic alterations in DDR pathways with previously published sequencing data. Mutations in DDR pathway genes are associated with human cancers (4, 44, 45). Although somatic mutations were observed in DDR pathway genes in ESCC, the mutation rate was low. Polymorphisms of BER pathway genes were reported to be associated with risk of ESCC (13), and frequent mutations of MMR pathway genes occurred in colorectal cancer and were associated with the etiology of colorectal cancer (45). However, no significant mutations in BER and MMR pathways were identified in ESCC. Interestingly, Two DSB repair pathways HR and NHEJ carried most gene mutations. Contrast to the gene mutation profile, CNVs especially amplification was observed to be the dominant alteration type in DDR pathways. Amplification of DDR pathway genes was reported to play a crucial role in cancer progression (9, 26, 46). We observed that obvious gene amplification occurred in multiple DDR pathways in ESCC. Similar to the gene mutation profile, HR and NHEJ had a significant gene amplification profile. Previous studies demonstrated that DSB repair pathway genes were over-expressed in cancers and high expression of these genes was associated with cancer development and resistance to chemotherapy and radiotherapy (8, 10, 11, 25, 26, 47, 48). However, how altered DSB repair pathways contribute to ESCC is much less explored. We found that amplification of DSB repair pathways was associated with poorer overall survival. Gain of MRN complex genes, *RAD54B* and *RAD51B*, whose alterations were reported to be involved in cancer progression (9, 47, 48), was related to poorer overall survival. The NHEJ pathway gene *PRKDC*, which is linked to the development of multiple cancers (10, 31, 49), was also significantly amplified in ESCC. Nevertheless, how amplification of these genes contributes to the development of ESCC is still unclear. CNVs are important factors that can affect gene expression (50). We observed that DSB repair pathway genes, especially the genes with notable amplification such as *RAD54B, RAD51B, NBS1* and *PRKDC*, were up-regulated in ESCC. GSVA result further showed that DSB repair pathways were obviously up-regulated in ESCC, and high pathway activities of DSB repair pathways were related to shorter overall survival and lymph node metastasis. These findings suggest that alterations in DSB repair pathways might play important roles in the development of ESCC.

Although radiotherapy is widely used for ESCC treatment, locoregional disease persists or recurs in 40 to 60% of patients owing to the ability of ESCC cells to become radio-resistant (51, 52). Thus, it is critical to well understand the underlying mechanisms of radio-resistance in ESCC and find the ways to improve the effectiveness of radiotherapy. As up-regulated DDR pathways confer therapeutic resistance in cancers, discovery and development of targeted agents that abrogate specific proteins in DDR pathways is a promising strategy for developing precise cancer treatments. Hitherto, many inhibitors that target specific DDR pathways have been developed (4). However, only a few DDR inhibitors have been used for ESCC treatment. (53–55). Whether targeting DSB repair is an effective strategy for ESCC treatment is much less explored. It is known that up-regulation of DSB repair pathway genes is one of the reasons for cancer radio-resistance. Therefore, targeting DSB repair pathways is a potential effective strategy to enhance radio-sensitivity (4–6, 8, 11). As one of the most famous examples of HR inhibitors, mirin was developed against endonuclease activity of MRE11 and used to effectively inhibit multiple cancers (32, 56, 57). Similarly, NU7441, a highly selective inhibitor for DNA-PK, blocked NHEJ of radiation-induced DSBs and enhanced cancer radio-sensitivity (33, 34, 58, 59). However, whether mirin and NU7441 could affect the radio-sensitivity of ESCC with DSB repair pathway up-regulation is still unclear. We made the first demonstration that combination of mirin and NU7441 with IR treatment significantly enhanced the radio-sensitivity of ESCC cells with DSB repair pathway gene amplification. This result provides a basis for exploring precision medicine strategies for ESCC treatment. Nevertheless, the effect of mirin and NU7441 on xenograft tumors in mice needs to be explored in the future.

In conclusion, this is the first report to comprehensively identify the alterations of DDR pathways in ESCC, and demonstrated that altered DSB repair pathway genes might contribute to ESCC progression. However, the molecular functions of these genes in ESCC should be further studied. We also firstly revealed two DSB repair pathway inhibitors mirin and NU7441 could obviously improve the radio-sensitivity of ESCC cells with DSB repair pathway gene amplification, showing the potential clinical application in ESCC treatment.

## Data Availability Statement

Publicly available datasets were analyzed in this study. This data can be found here: European Genome-phenome Archive (https://www.ebi.ac.uk/ega/, accession number EGAS00001000709), NCBI Sequence Read Archive (https://www.ncbi.nlm.nih.gov/sra, accession number SRA112617) and Gene Expression Omnibus (https://www.ncbi.nlm.nih.gov/geo/, accession number GSE54995 and GSE53624). Additionally, gene mutation and clinical information within this study can be found in the supplementary materials of the previously published studies (https://www.nature.com/articles/nature13176) (https://www.cell.com/ajhg/fulltext/S0002-9297(15)00100-7).

## Ethics Statement

Our research was approved by the Ethics Committee of Cancer Hospital Chinese Academy of Medical Sciences and Peking Union Medical College.

## Author Contributions

GW collected, analyzed, interpreted data, performed experiments, and wrote the manuscript. SG interpreted data and provided support. WZ, ZL, JX, DL, and YW provided supervision and support. QZ conceived the concept, designed the study, wrote the manuscript, and took responsibility for the whole manuscript. All authors contributed to the article and approved the submitted version.

## Funding

This work was supported by the National Natural Science Foundation of China (81830086 and 81988101), Beijing Municipal Commission of Health and Family Planning Project (PXM2018_026279_000005).

## Conflict of Interest

The authors declare that the research was conducted in the absence of any commercial or financial relationships that could be construed as a potential conflict of interest.
